# Aberrant expression of a five‐microRNA signature in breast carcinoma as a promising biomarker for diagnosis

**DOI:** 10.1002/jcla.23063

**Published:** 2019-10-08

**Authors:** Amirreza Bitaraf, Sadegh Babashah, Masoud Garshasbi

**Affiliations:** ^1^ Department of Molecular Genetics Faculty of Biological Sciences Tarbiat Modares University Tehran Iran; ^2^ Department of Medical Genetics Faculty of Medical Sciences Tarbiat Modares University Tehran Iran

**Keywords:** biomarker, breast cancer, diagnosis, HER‐2, microRNA

## Abstract

**Background:**

Breast cancer (BC) is the most common malignancy among females with dismal quality of life in patients. It has been proven that epigenetic factors, especially microRNAs, are involved in breast carcinogenesis and progression. This study aimed to assess the expression and clinical performances of a five‐microRNA signature (miR‐127‐3p, miR‐133a‐3p, miR‐155‐5p, miR‐199b‐5p, and miR‐342‐5p) in breast cancer and adjacent normal tissues to identify a potential biomarker for BC and investigate the relationship between their expression and clinicopathological features of BC patients as well.

**Methods:**

In this case‐control investigation, we recruited 50 pairs of tumor and matched non‐tumor surgical specimens from patients diagnosed with BC. Expression levels of miR‐127‐3p, miR‐133a‐3p, miR‐155‐5p, miR‐199b‐5p, and miR‐342‐5p were measured in BC and adjacent normal tissues by RT‐qPCR.

**Results:**

We found that miR‐127‐3p, miR‐133a‐3p, miR‐199b‐5p, and miR‐342‐5p were significantly down‐regulated, while miR‐155‐5p was significantly up‐regulated in BC tumor tissues compared with the corresponding adjacent normal tissues. The decreased expression of miR‐127‐3p, miR‐133a‐3p, miR‐342‐5p, and up‐regulation of miR‐155‐5p showed a significant correlation with disease stage. We also found a significant down‐regulation of miR‐127‐3p, miR‐199b‐5p, and miR‐342‐5p compared in HER‐2‐negative patients. Our results indicated that miR‐155‐5p had a higher expression level in HER‐2‐positive patients. Receiver operating characteristic (ROC) curve analysis demonstrated that all these five microRNAs can serve as potential biomarkers to distinguish between tumor and non‐tumor breast tissue samples.

**Conclusions:**

The present findings suggested that dysregulation of this five‐miRNA signature might be considered as a promising and functional biomarker for BC diagnosis.

## 1 INTRODUCTION

Breast cancer (BC) is the most commonly diagnosed cancer among women worldwide. Although early detection techniques and therapy methods have been improved, BC is still the leading cause of cancer death in females, and it is estimated that 627 000 women died from BC in 2018.^1^, ^2^ BC starts as a local disease but it might probably metastasize to distant organs such as bone, lung, regional lymph nodes, liver, and brain. Accordingly, early diagnosis is crucial to select the most appropriate treatment for patients with BC.^3^ Mammography is the gold standard screening method in BC which helps to find early signs of BC. Furthermore, other imaging techniques such as MRI, CT scan, PET scan, and elastography could be utilized as beneficial methods to detect BC. Nevertheless, all these tools have their own limitations including being expensive, radiation risks, and more importantly, lack of specificity which count them as inefficient screening methods.^4^, ^5^ In this regard, numerous efforts have been strived to find better diagnostic and therapeutic tools for patients with BC. By having high sensitivity, being noninvasive, and more specificity, biochemical biomarkers, including proteins, DNAs, microRNAs (miRNAs, miRs), and long non‐coding RNAs (lncRNAs), are recently considering as easily accessible markers with promising potentials for the detection of BC at early stages.^6^, ^7^

miRNAs are a class of small non‐coding RNAs with a length of about 22 nucleotides which regulate gene expression at the post‐transcriptional level.^8^ miRNAs are the key player of multiple crucial biological processes such as proliferation, differentiation, and apoptosis. Unsurprisingly, aberration in miRNAs expression has been demonstrated in various types of cancers. miRNAs may play a crucial role a class of oncogenes or tumor suppressor genes in cancer.^9^, ^10^, ^11^ In BC, miRNA profiling has allowed for the identification of biomarker signatures associated with the diagnosis, staging, progression, and prognosis.^12^, ^13^, ^14^, ^15^, ^16^

The aim of the present study was to analyze the expression levels of miR‐127‐3p, miR‐133a‐3p, miR‐155‐5p, miR‐199b‐5p, and miR‐342‐5p in BC patient tissues and normal counterparts. Furthermore, we investigated the correlation between alterations of these miRNAs with clinical phenotypes and HER‐2 expression status of the patients.

## 2 MATERIALS AND METHODS

### 2.1 Clinical samples

Formalin‐fixed paraffin‐embedded (FFPE) tissues of the present study were collected from the tumor bank of Imam Khomeini Cancer Institute, Tehran, Iran. These tissues were diagnosed through the assessment of histopathological parameters according to the World Health Organization (WHO) criteria for the histologic grade, the TNM system for stage classification and HER‐2 status. Written informed contentment was obtained from all patients, and the study was approved by the ethics committee at participating center. A total of 50 tumor samples and 50 matched non‐tumor samples from the same patients were recruited to this study. A record of clinicopathological parameters including tumor stage, lymph node metastasis, hormonal receptor status, and HER‑2 status for these tissue samples are summarized in Table 1.

**Table 1:** The clinicopathological characteristics of breast cancer patients

Characteristics	No.	%
Age (y)		
<50	17	34
≥50	33	66
Tumor size (cm)		
<3	18	36
≥3	32	64
Primary tumor (T stage) (cm)		
T1: ≤2.0	15	30
T2: >2‐≤5	24	48
T3: >5	11	22
Regional lymph nodes (N stage)		
NX	5	10
N0	13	26
N1	11	22
N2	16	32
N3	5	10
Distant metastasis (M stage)		
MX	5	10
M0	17	34
M1	28	56
Lymph node metastasis		
Negative	17	34
Positive	33	66
Tumor stage		
I + II	27	54
III	23	46
Estrogen receptor status		
Negative	15	30
Positive	31	62
Unknown	4	8
Progesterone receptor status		
Negative	18	36
Positive	29	58
Unknown	3	6
HER‐2 status		
Negative	22	44
Positive	27	54
Unknown	1	2

### 2.2 RNA isolation and quality evaluation

Samples were washed multiple times with xylene to solubilize and remove the paraffin, and total RNA was extracted from FFPE tissues using TRIzol (Invitrogen). In the way, the concentration of total RNAs was measured by NanoDrop 2000c (Thermo Fisher Scientific), and the integrity of RNA samples was confirmed by 2% gel electrophoresis. To remove the possible little amount of genomic DNA, the samples were treated with RNase‐free DNase.

### 2.3 cDNA synthesis and RT‐qPCR

Poly‐(A)‐tailing and complementary DNA (cDNA) synthesis were performed by reverse transcription of 1 μg total RNA using MiR‐Amp Kit (ParsGenome). The anchored oligo(dT) sequence for cDNA synthesis was as follows: GCGTCGACTAGTACAACTCAAGGTTCTTCCAGTCACGACGTTTTTTTTTTTTTTTTTT[N]. Mature miRNA expression was measured by miRNA‐specific primer and miRNA RT‐qPCR master mix kit (ParsGenome) on an ABI StepOne Sequence Detection System (Applied Biosystems) with the following conditions: an initial denaturation at 95°C for 5 minutes, 40 cycles of denaturation at 95°C for 10 seconds, and annealing/extension at 60°C for 30 seconds. Also, each cycle of proliferation stage was completed by a separation step included 95°C for 15 seconds, 60°C for 30 seconds, and 90°C for 15 seconds to further analyze the melting curve. The relative expression of each miRNA was normalized to U48 snRNA as an internal control and calculated using the 2^−ΔΔCt^ method.^17^, ^18^ Primer sequences for U48 snRNA were as follows: U48‐Forward: TGACCCCAGGTAACTCTGAGTGTGT; Universal‐Reverse: GCGTCGACTAGTACAACTCAAG.

### 2.4 Statistical analysis

Data analyses were performed using ABI StepOne Real‐Time PCR Software v2.0.2 (Applied Biosystems), and figures were made using GraphPad Prism v.7.0 (GraphPad Software). The results were analyzed by performing *t* tests in which *P* < .05 considered statistically significant. To be on the safe side, all experiments were carried out at least two times.

## 3 RESULTS

### 3.1 Expression analysis of a five‐miRNA signature in tumor and non‐tumor samples

We utilized bioinformatics tools for mining miRNAs, which aim at identifying the most possible miRNAs that potentially cause BC development. Furthermore, we reviewed previous experimental findings that suggested possible role of these miRNAs in BC progression. Finally, five miRNAs including miR‐127‐3p, miR‐133a‐3p, miR‐155‐5p, miR‐199b‐5p, and miR‐342‐5p were selected to be evaluated as possible biomarkers in BC patients. The reaction efficiencies for each primer set were calculated on 5‐fold serial dilutions of cDNA samples. As shown in Figure S1 and Table S2, the amplification efficiencies of our candidate miRNAs and housekeeping gene were approximately equal with high linear correlation, indicating the validity of the assay for relative expression quantification. Moreover, the uniqueness and specificity of the amplified products were confirmed by dissociation curve analysis. The presence of single and sharp melting curves confirmed that no primer‐dimer or non‐specific products were generated during the amplification reaction (Figure S2).

**Figure 1:**
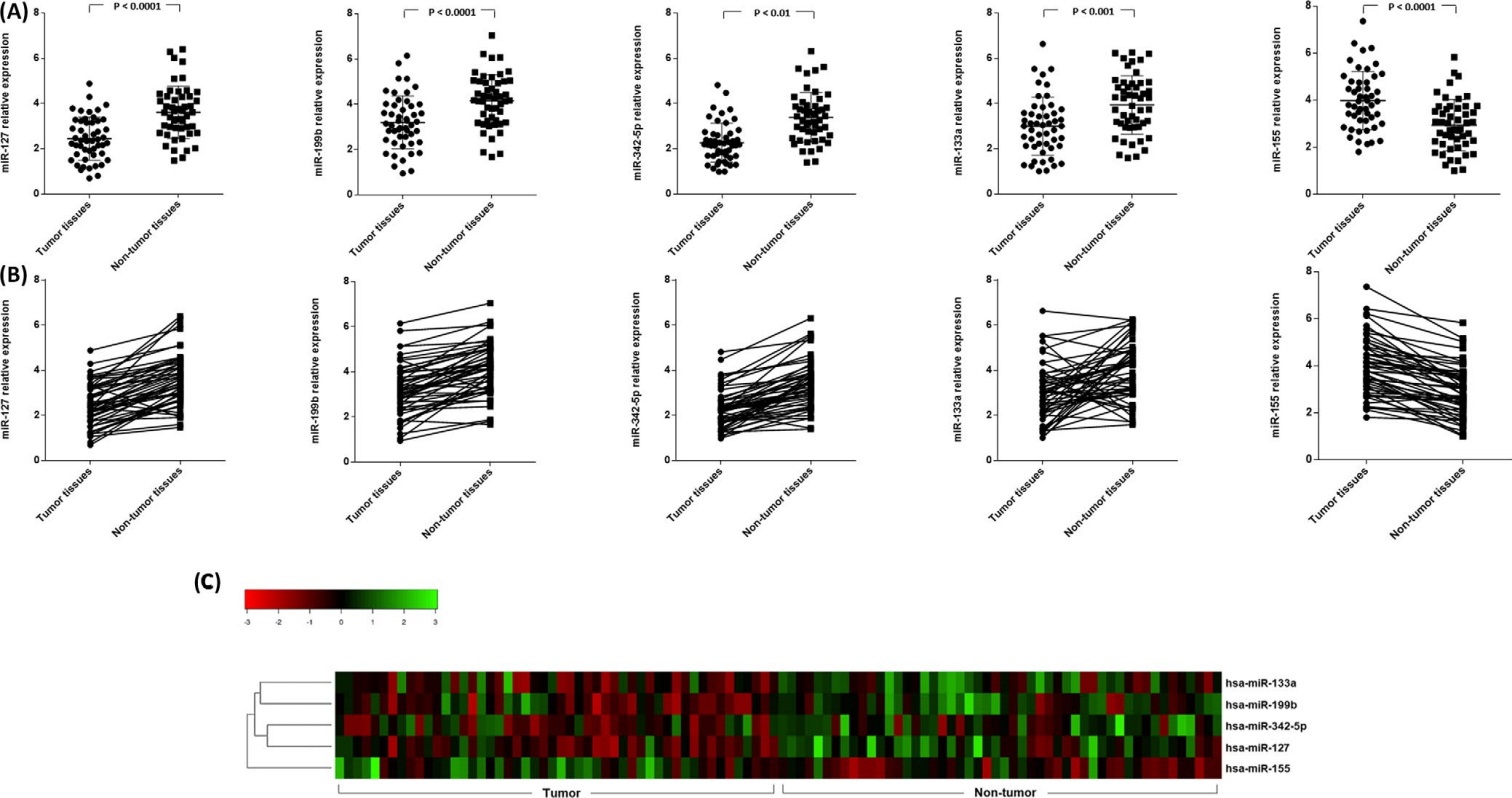
Expression of microRNAs in tumor and non‐tumor breast cancer patients. A, B, microRNAs expression profile indicated a significant down‐regulation of miR‐127 (0.677‐fold, *P*‐value < .0001), miR‐199b (0.771‐fold, *P*‐value < .0001), miR‐342‐5p (0.784‐fold, *P*‐value = .0017), and miR‐133a (0.758‐fold, *P*‐value = .0003), in tumor compared with non‐tumor breast cancer tissues. Meanwhile, the expression of miR‐155 (1.357‐fold, *P*‐value < .0001) was significantly lower in tumor tissues than that of non‐tumor tissues obtained from the same patients. C, Unsupervised hierarchical clustering analysis using the differentially expressed miRNAs separate tumor and non‐tumor breast tissues. The heatmap (Euclidian distance, complete linkage) represents miRNAs with high expression in green and miRNAs with low expression in red

To measure the expression status of our candidate miRNAs in BC, a RT‐qPCR assay was conducted to determine the expression of miR‐127‐3p, miR‐133a‐3p, miR‐155‐5p, miR‐199b‐5p, and miR‐342‐5p in 50 pairs of BC and corresponding non‐tumor tissue samples. Results revealed that there was a significant down‐regulation of miR‐127‐3p (0.677‐fold, *P*‐value < .0001), miR‐199b‐5p (0.771‐fold, *P*‐value < .0001), miR‐342‐5p (0.784‐fold, *P*‐value = .0017), and miR‐133a‐3p (0.758‐fold, *P*‐value = .0003) in tumor samples compared to those of their matched non‐tumor controls. On the other hand, we found that miR‐155‐5p was significantly up‐regulated (1.357‐fold, *P*‐value < .0001) in tumor samples in comparison with those of their matched non‐tumor controls (Figure 1A,B). Unsupervised hierarchical clustering analysis of the relative expression of the differentially expressed miRNAs revealed that this set of markers is able to discriminate between the tumors and the non‐tumor breast tissues (Figure 1C).

### 3.2 Correlation of miRNAs expression levels with level of malignancy and HER‐2 status in human BC

**Figure 2:**
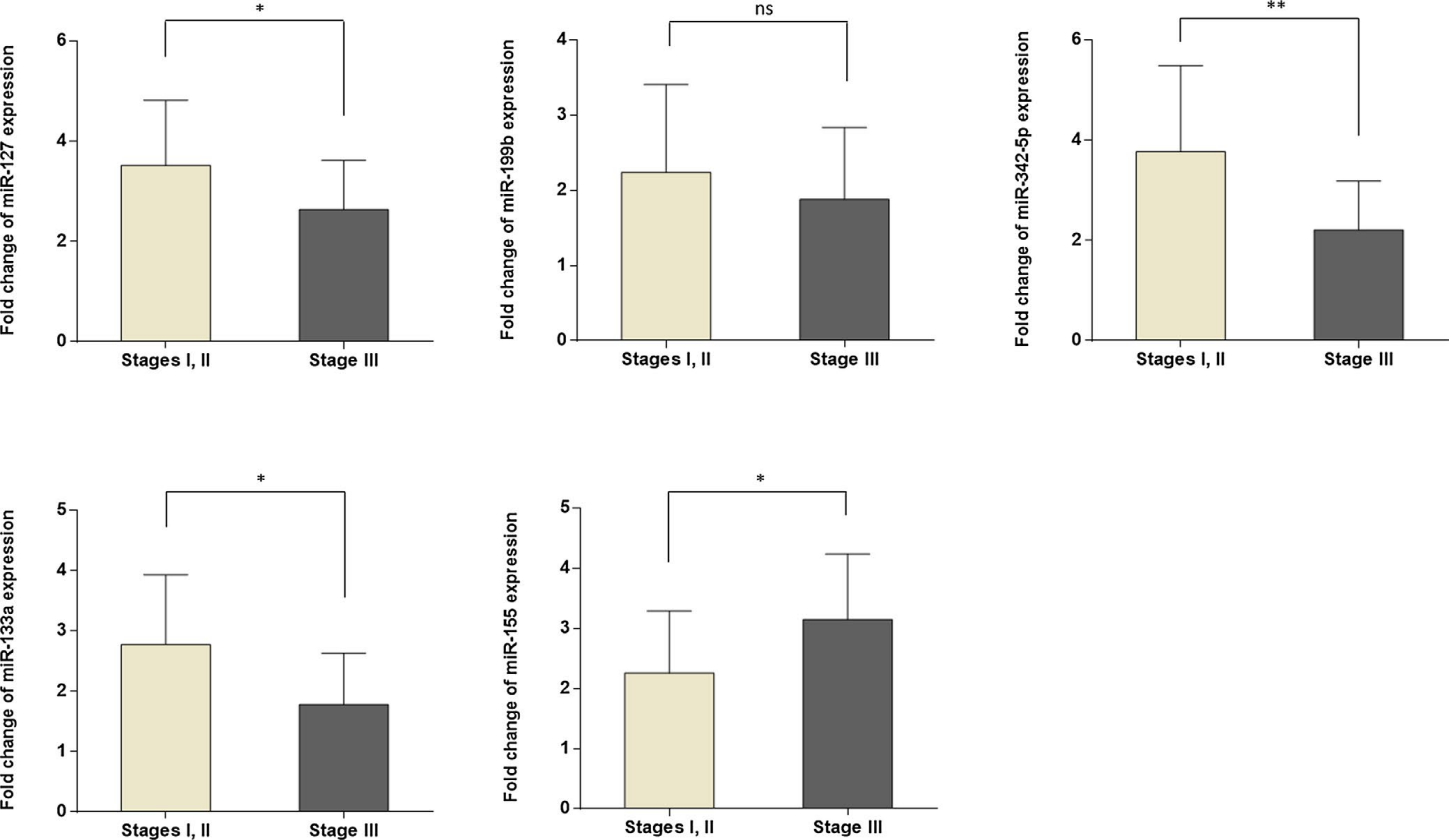
Comparison of miRNA expression level in breast cancer patients with different tumor stages. Note that the expression of miR‐127, miR‐133a, and miR‐342‐5p was significantly lower in higher stage. The observed difference in the expression level of miR‐199b was not statistically significant. On the other hand, miR‐155 was significantly up‐regulated in stage III in comparison with stages I and II

We analyzed whether the expressional evaluation of this five‐miRNA signature was correlated with clinicopathological characteristics of BC patients. As compared with the different stages of malignancy of BC, the expression levels of miRNAs including miR‐127‐3p, miR‐133a‐3p, and miR‐342‐5p were significantly lower in stages III compared with stages I and II, except miR‐199b‐5p which down‐regulated non‐significantly in higher stages, whereas the miR‐155‐5p was significantly up‐regulated in stages III in comparison with stages I and II (Figure 2).

**Figure 3:**
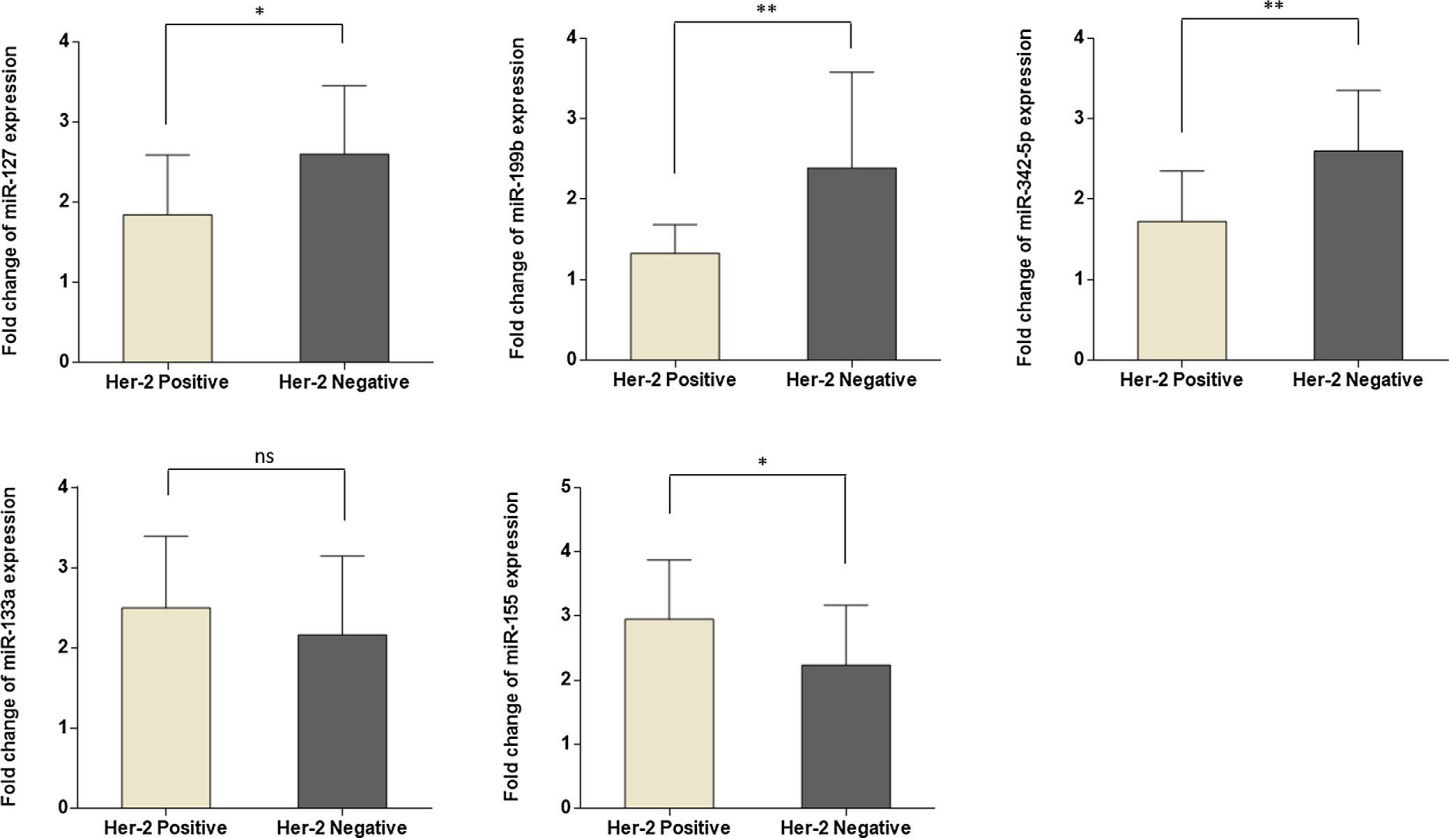
microRNA expression level fold changes based on HER‐2 status. Patients who are HER‐2‐positive showed significant down‐regulation of miR‐127, miR‐199b, and miR‐342‐5p, compared with HER‐2‐negative patients. Both miR‐133a and miR‐155 have higher expression in HER‐positive patients, although the high expression of miR‐133a was not significant

In this study, we also investigated the possible association between expression levels of miRNAs and HER‐2 status in BC tissues. The expression levels of miR‐127‐3p, miR‐199b‐5p, and miR‐342‐5p were significantly lower, while the miR‐133a‐3p showed a non‐significant up‐regulation in HER‐2‐positive patients compared to patients who are HER‐2‐negative. Moreover, miR‐155‐5p was up‐regulated in HER‐2‐positive patients which indicated a significant difference between HER‐2‐positive and HER‐2‐negative patients (Figure 3).

### 3.3 Determination of the biomarker quality for BC

**Figure 4:**
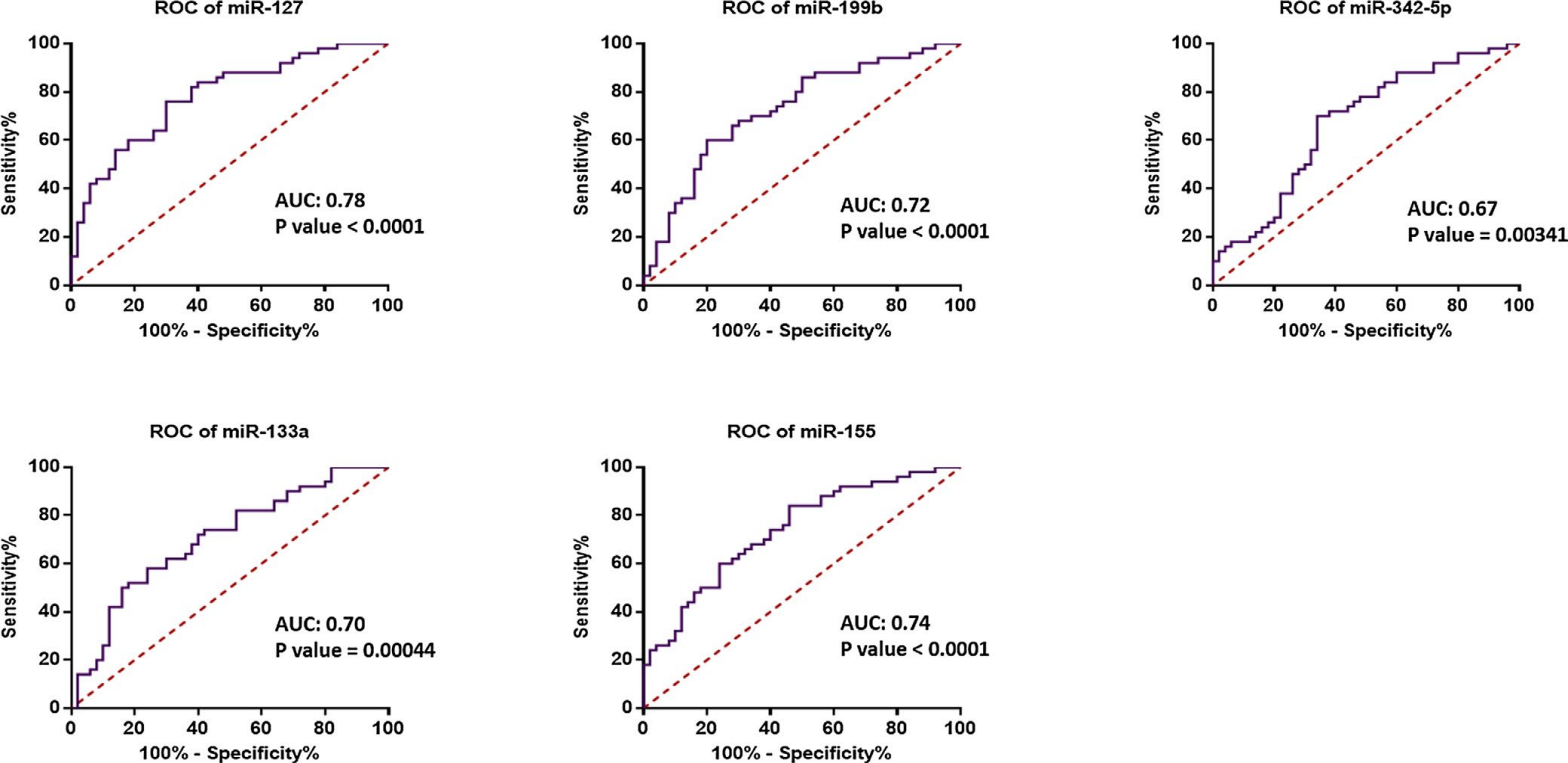
ROC curve analyses were performed to discriminating tumor from non‐tumor breast tissue samples. ROC curve and its calculated area under curve (AUC) of miR‐127 (78%), miR‐199b (72%), miR‐342‐5p (67%), miR‐133a (70%), and miR‐155 (74%) suggest that all of these five miRNAs may be appropriate tumor biomarkers for breast cancer

We utilized ROC curve analysis to evaluate the sensitivity and specificity of the expression levels of these miRNAs to discriminate BC tissues from healthy tissues. The calculated area of the miRNAs which are examined in this study suggests that all of these five miRNAs may be suitable as a tumor marker and can potentially serve as an efficient diagnostic biomarker for BC (Figure 4).

## 4 DISCUSSION

Breast cancer is the most prevalent cancer among women worldwide.^19^ Numerous studies have been reported that miRNAs have regulatory functions in pathological processes, in particular in the development and progression of tumors.^9^ Hence, it is possible that many miRNAs could serve as biomarkers for the diagnosis of different types of cancer, including BC.^16^

Integrative computational bioinformatics approaches have been utilized as an effective tool to detect the potential outlier miRNAs in cancer.^20^, ^21^ Furthermore, as a rational approach, preliminary detection of candidate miRNAs derived from large‐scale expression profiling data and low‐throughput experimental verification for the selected outlier miRNAs can be used to choose candidate miRNAs.^22^, ^23^ In the present study, according to literature reviews and data mining for miRNAs involved in the pathogenesis of breast cancer, we selected the candidate miRNAs; to begin with, the candidate miRNAs not only should have been highly dysregulated in human BC but also they must have been easily detectable. As the second criteria used, candidate miRNAs must have unambiguously annotated in miRBase (Release 22.1), and last but not least, the candidate miRNAs should have been involved in signaling pathways which contribute to the pathogenesis of BC. According to these criteria, the candidate miRNAs including miR‐127‐3p, miR‐133a‐3p, miR‐155‐5p, miR‐199b‐5p, and miR‐342‐5p were culled to be analyzed and confirmed by RT‐qPCR.

In this study, a comparison between the expression profile of our candidate miRNAs in breast carcinoma tissue samples and their normal tumor margin was applied. In other words, this study has been applied to investigate whether the candidate miRNAs have the potential to be a novel diagnostic marker. Derived results showed a significant down‐regulation in miR‐127‐3p, miR‐133a‐3p, miR‐199b‐5p, and miR‐342‐5p in tumor tissues of the patients compared with the normal margin tissues, while miR‐155‐5p has been up‐regulated in the aforementioned samples. Interestingly, a correlation between the expression levels of these miRNAs with some clinical characteristics of disease was observed. Collectively, these results suggest that these microRNAs signature might be considered as a tool for early diagnosis in BC.

Aberrant expression of miR‐127 is reported in several cancer types such as hepatocellular carcinoma, ovarian carcinoma, and gastric cancer.^24^, ^25^, ^26^ miR‐127 is a tumor suppressor which regulates BC cell proliferation and senescence by targeting *BCL6*. Chen et al^27^ showed that miR‐127 was down‐regulated and its lower expression leads to the up‐regulation of *BCL6* in BC tissues. Wang et al^28^ showed that miR‐127 was down‐regulated in the BC tissues compared to noncancerous tissues. However, there was no significant correlation between miR‐127 expression and HER‐2 status. Our data confirmed the previous findings of Wang et al. Meanwhile, our results indicated that miR‐127‐3p expression is significantly down‐regulated in HER‐2‐positive patients.

miR‐133 family is a group of miRNAs which contains three members, including miR‐133a‐1, miR‐133a‐2, and miR‐133b.^29^ Studies which held by Wu et al^30^ demonstrated that low expression of miR‐133a was correlated with the poor survival of BC patients and restoration of miR‐133a expression inhibited BC cell growth and invasion. Sui et al^31^ also confirmed these findings, and they reported that miR‐133a levels were significantly decreased in BC patients which is associated with high levels of the malignancy, lymph node metastasis, and shorter survival time of the patients. Although miR‐133a has been reported to be a tumor suppressor and down‐regulated in several cancer types, Shen et al^32^, ^33^, ^34^ observed an up‐regulation of circulating miR‐133a in BC patients. We found a down‐regulation of miR‐133a‐3p in BC tissues in comparison with normal counterparts, and this aberrant expression is significantly associated with clinical stage.

Several studies have shown that miR‐155 is an oncogene which regulates several cancer cell process, including cell proliferation, migration, invasion, metastasis, and epithelial‐mesenchymal transition (EMT).^35^, ^36^, ^37^ Khaleghifard et al^4^ revealed that miR‐155 is decreased in plasma and tissue of BC patients compared with control ones. Their findings also demonstrated that common treatment BC strategies such as operation, chemotherapy, and radiotherapy causes down‐regulation of miR‐155. Similar to the previous findings, our findings indicated that miR‐155‐5p acts as an oncogene and up‐regulated in BC patients. Our results also showed that high expression of miR‐155‐5p is correlated with clinical stage and HER‐2 status in BC patients.

It has been shown that miR‐199b acts as a tumor suppressor in BC. In triple negative breast cancer (TNBC) cells, miR‐199b suppresses cell proliferation and invasion by directly targeting DDR1.^38^ BC patients who showed lower expression level of miR‐199b had poorer overall survival rate than those with high level. Furthermore, it is indicated that low expression of miR‐199b is correlated with poor prognosis, advanced TNM stage, and positive lymph node metastasis in BC.^39^ Our findings demonstrated that miR‐199b‐5p is down‐regulated in BC tissues compared with non‐tumor counterparts, and deregulation of miR‐199b‐5p is correlated with clinical stage and HER‐2 status.

A research by Savad et al^40^ on various subtypes of BC demonstrated that miR‐342 was down‐regulated in BC patients and may be used as a potential biomarker for the diagnosis of TNBC. Lindholm et al^41^ suggested that miR‐342 is associated with the cell proliferation rate of HER‐2‐positive breast cancer cells. Ectopic overexpression of miR‐342 suppressed two HER‐2 downstream pathways including ERK/MAPK and SAPK/JNK which results in cell proliferation inhibition. Considering the role of miR‐342 as a regulator of the HER‐2 pathway, we determined to evaluate the expression of miR‐342‐5p in BC patients. Our findings showed a significant correlation between low expression of miR‐342‐5p and HER‐2 status in BC patients.

Our study was set to evaluate the diagnostic role of miR‐127‐3p, miR‐133a‐3p, miR‐155‐5p, miR‐199b‐5p, and miR‐342‐5p expression in the cancerous and normal tissues of the patients with BC. We demonstrated that miR‐127‐3p, miR‐133a‐3p, miR‐199b‐5p, and miR‐342‐5p were down‐regulated; however, miR‐155‐5p represented up‐regulation in tumor samples in comparison with those of their matched non‐tumor controls. The expression level of these miRNAs was associated with the extensiveness of the malignancy and HER‐2 expression status. In addition, the results revealed that miR‐127‐3p, miR‐199b‐5p, miR‐342‐5p, miR‐133a‐3p, and miR‐155‐5p could serve as valuable biomarkers for the diagnosis of BC with AUCs of 0.78, 0.72, 0.67, 0.70, and 0.74, respectively.

One of the most important points for an effective prognosis and diagnosis approach is to identify early‐stage BC, and our study suggested that evaluating the expression levels of miR‐127‐3p, miR‐133a‐3p, miR‐155‐5p, miR‐199b‐5p, and miR‐342‐5p might be clinically useful in BC patients. Our results confirmed the previous findings which showed that aberrant expression of these five miRNAs can improve cancerous growth.^27^, ^30^, ^35^, ^39^, ^41^ Altogether, dysregulation of this five‐miRNA signature might be used as a promising biomarker in detecting and following the disease progression in BC patients.
