# Supplementary material for: Aberrant expression of a five‐microRNA signature in breast carcinoma as a promising biomarker for diagnosis
Source: J Clin Lab Anal. 2019 Oct 8;34(2):e23063. doi: 10.1002/jcla.23063 (PMC7031575; doi:10.1002/jcla.23063)
Supplement: Supplementary file 1 [file JCLA-34-e23063-s001.docx]

**Supplementary data**

**Supplementary Table S1: microRNA target sequence and its accession no. measured in this study according to miRBase 22.1**

| **microRNA name** | **Target sequence** | **Target sequence accession** |
| --- | --- | --- |
| hsa-miR-127-3p | UCGGAUCCGUCUGAGCUUGGCU | MIMAT0000446 |
| hsa-miR-133a-3p | UUUGGUCCCCUUCAACCAGCUG | MIMAT0000427 |
| hsa-miR-155-5p | UUAAUGCUAAUCGUGAUAGGGGUU | MIMAT0000646 |
| hsa-miR-199b-5p | CCCAGUGUUUAGACUAUCUGUUC | MIMAT0000263 |
| hsa-miR-342-5p | AGGGGUGCUAUCUGUGAUUGA | MIMAT0004694 |

**Supplementary Table S2: Slope and efficiencies of standard curves for each primer set**

| **Gene** | **Slope** | **Efficiency** |
| --- | --- | --- |
| hsa-miR-127-3p | -3.5243 | 0.92 |
| hsa-miR-133a-3p | -3.3757 | 0.98 |
| hsa-miR-155-5p | -3.3086 | 1.01 |
| hsa-miR-199b-5p | -3.4971 | 0.93 |
| hsa-miR-342-5p | -3.2986 | 1.01 |
| U48 snRNA | -3.2429 | 1.03 |

**
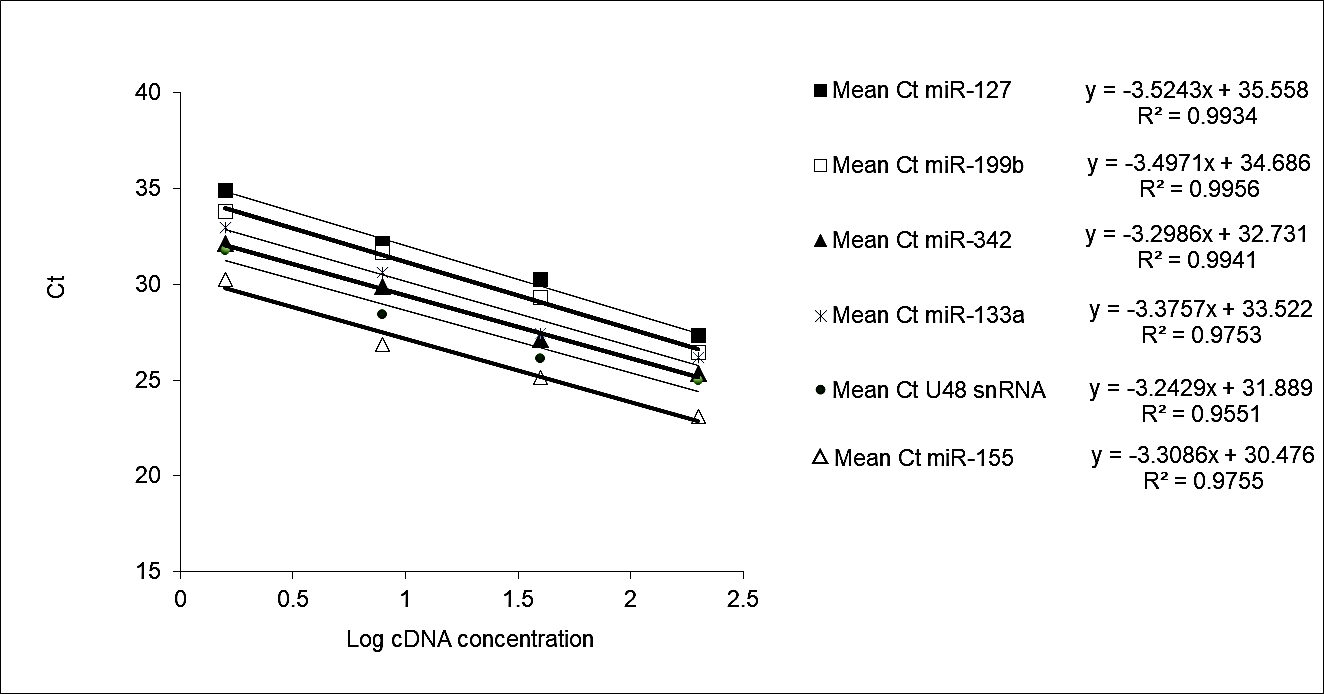
**

**Supplementary Fig. S1: RT-qPCR efficiencies.** To determine the amplification efficiency, standard curves via plotting the logarithmic amount of serially diluted cDNA input against the corresponding Ct values was exploited. The efficiency (E) of RT-qPCR was calculated according to the slope of the standard curve and the following equation: E = 10^[-1/slope]^. All slopes were approximately equal with high linear correlation.


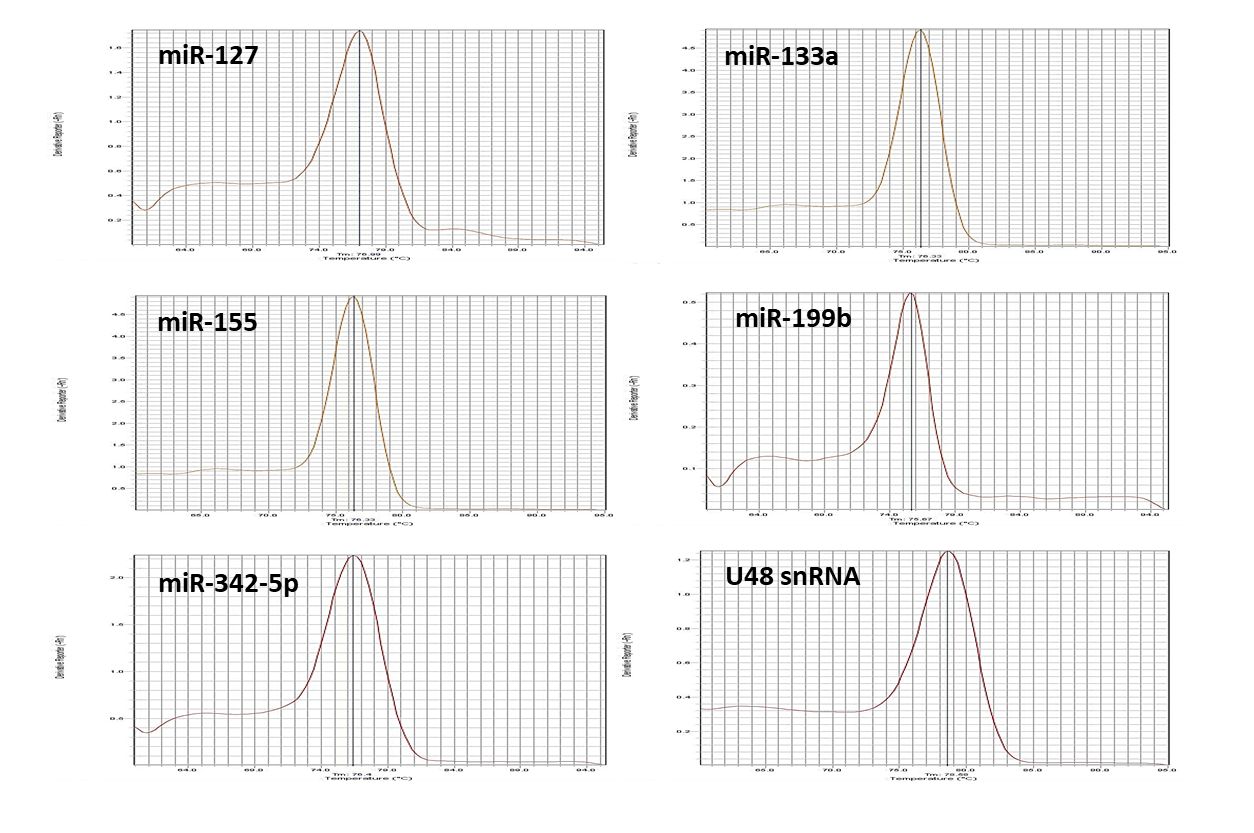


**Supplementary Fig. S2: Uniqueness and specificity of the RT-qPCR products.** Dissociation curve analysis performed on PCR products obtained from amplification reactions for miR-127, miR-133a, miR-155, miR-199b, miR-342-5p, 5s rRNA. The curves featured by a single and sharp peak at expected Tm.
